# Assessing the survival time of hospitalized patients in Eastern Ethiopia during 2019–2020 using the Bayesian approach: A retrospective cohort study

**DOI:** 10.1002/hsr2.2135

**Published:** 2024-05-28

**Authors:** Chalachew Gashu, Yoseph Kassa, Habtamu Geremew, Mengestie Mulugeta

**Affiliations:** ^1^ Department of Statistics College of Natural and Computational Science, Oda Bultum University Chiro Ethiopia; ^2^ Department of Nursing College of Health Science, Oda Bultum University Chiro Ethiopia; ^3^ Department of Physiotherapy Tikur Anbessa Hospital Addis Ababa Ethiopia

**Keywords:** Bayesian analysis, severe acute malnutrition, survival data analysis, survival time

## Abstract

**Background and Aims:**

Severe acute malnutrition remains a significant health challenge, particularly in low‐ and middle‐income countries. The aim of this study was to determine the survival time of under‐five children with severe acute malnutrition.

**Methods:**

A retrospective cohort study was conducted at a hospital, focusing on under‐five children with severe acute malnutrition. The study included 322 inpatients admitted to the Chiro hospital in Chiro, Ethiopia, between September 2019 and August 2020, whose data was obtained from medical records. Survival functions were analysed using Kaplan‒Meier plots and log‐rank tests. The survival time of severe acute malnutrition was further analysed using the Cox proportional hazards model and Bayesian parametric survival models, employing integrated nested Laplace approximation methods.

**Results:**

Among the 322 patients, 118 (36.6%) died as a result of severe acute malnutrition. The estimated median survival time for inpatients was found to be 2 weeks. Model selection criteria favored the Bayesian Weibull accelerated failure time model, which demonstrated that age, body temperature, pulse rate, nasogastric (NG) tube usage, hypoglycemia, anemia, diarrhea, dehydration, malaria, and pneumonia significantly influenced the survival time of severe acute malnutrition.

**Conclusions:**

This study revealed that children below 24 months, those with altered body temperature and pulse rate, NG tube usage, hypoglycemia, and comorbidities such as anemia, diarrhea, dehydration, malaria, and pneumonia had a shorter survival time when affected by severe acute malnutrition under the age of five. To reduce the death rate of children under 5 years of age, it is necessary to design community management for acute malnutrition to ensure early detection and improve access to and coverage for children who are malnourished.

## 1 INTRODUCTION

The phrase “today's kids are tomorrow's world” or “tomorrow's dad” resonates with deep concern worldwide. However, many children across the globe still lack access to basic amenities. A crucial determinant of a child's health is their dynamic process of body size growth.^1^ Nutrition plays a significant role in determining overall health and is essential for survival, a high standard of living, and general well‐being. Malnutrition, characterized by an imbalance or insufficiency of essential nutrients, can lead to various pathological conditions.^2^ Severe acute malnutrition (SAM) is categorized based on the degree of wasting and mid‐upper‐arm circumference, with SAM representing significant wasting (low weight for height, i.e., below −3 *z* score) or a low mid‐upper arm circumference (MUAC < 11.5 cm). SAM is one of the most pressing health issues globally, particularly in middle‐ and low‐income countries. It affects 52 million children under the age of five, with 17 million experiencing critical malnutrition. More than half of all lost children worldwide are found only in Southern Asia and Sub‐Saharan Africa. In Africa, 14 million children under the age of five are wasted, with 4.1 million being severely wasted.^3^ The risk of death for children experiencing SAM is nine times greater than that of children who receive adequate nutrition. Additionally, malnourished children are more susceptible to infections such as pneumonia and diarrhea, indirectly contributing to increased mortality rates.^3^ Ethiopia ranks second among sub‐Saharan countries in terms of the high prevalence of malnutrition.^4^ Previous studies conducted in Ethiopia have linked deaths among children affected by SAM to various factors, including anemia, respiration rate, vitamin A deficiency, diarrhea, skin dermatosis, age, body temperature, pulse rate, heart failure, mid‐upper‐arm circumference, pneumonia, hypoglycemic, dehydration, malaria, poor adherence to therapy regimens, and compromised vital signs.^4^, ^5^, ^6^, ^7^, ^8^ The management of acute malnutrition in Ethiopia involves the admission of above 25,000 children with SAM to hospitals each month. Failure to adequately diagnose and treat these children puts them at risk of mortality.^9^ To address the high mortality rate among children under the age of five suffering from SAM, the Ethiopian Ministry of Health has implemented inpatient therapeutic feeding programs in medical facilities. However, despite the availability of SAM management at hospitals and health centers nationwide, SAM‐related mortality rates remain significant, and there is limited knowledge about the recovery period and associated factors, especially in young children admitted to inpatient therapeutic feeding clinics.^10^

While previous studies in Ethiopia have focused on predictors of treatment outcomes, determinants of food security, and survival status using logistic regression analysis, descriptive and econometric methods, and survival analysis,^11^, ^12^ these studies do not account for critical factors such as shock and nasogastric tube usage, which greatly impact the survival status of children under the age of five. Additionally, logistic regression does not consider censoring of observations, which is crucial for time‐to‐event data analysis. Furthermore, the survival rate of hospital patients cannot be adequately assessed using logistic regression alone. Previous medical investigations have used Cox regression models along with other parametric models, such as Weibull, log‐logistic, exponential, and log‐normal models, to study the survival distribution of SAM.^13^, ^14^ Parametric survival models, such as AFT models, including Weibull, exponential, log‐normal, and log‐logistic, offer a more grounded understanding and provide meaningful insights compared to the Cox proportional hazards (Cox‐PH) model.^14^ In Bayesian survival analysis, parametric survival models play a crucial role, as they leverage previous studies' knowledge andminimize confounding by incorporating prior probability distributions.^15^, ^16^ The Bayesian approach integrates data with unbiased prior knowledge, providing a powerful means of analysis.^17^ However, the computational challenges and convergence issues associated with Markov chain Monte Carlo (MCMC) techniques pose limitations.^15^ Fortunately, by using Laplace approximations and sophisticated numerical techniques that take advantage of sparse matrices, the Bayesian methodology in conjunction with the integrated nested Laplace approximation (INLA) method provides a quicker and more accurate approximation of posterior marginal distributions.^16^ Given the prominence of SAM as a healthcare issue in countries with hospital‐based care and the existing research gaps, this study aims to analyse the SAM data set in Ethiopia using Bayesian parametric survival models and the INLA approach. The objectives include identifying prognostic markers in SAM, selecting the most suitable parametric survival models for the SAM data set, estimating the survival time of children under the age of five with SAM admitted to therapeutic feeding units, and evaluating Bayesian accelerated failure time models using the INLA approach. The findings of this study will not only contribute to understanding the factors influencing the survival time of children with SAM but also help raise public awareness about the causes of SAM‐related mortality. Additionally, the results can be shared with the Ethiopian Ministry of Health to aid policymakers in increasing public knowledge about the risk factors for SAM‐related death, which can be prevented and treated with early detection and appropriate care.

## 2 METHODOLOGY

### 2.1 Data description

#### 2.1.1 Study area

The journal's study location was Chiro Hospital, which is situated 325 kilometers east of Addis Ababa, the capital of Ethiopia.

#### 2.1.2 Target population

The target population for this study consists of all children under 5 years of age who have been registered in the therapeutic feeding unit (TFU) register book in the pediatric ward of Chiro General Hospital and diagnosed with SAM.

#### 2.1.3 Study population

The study population included all under 5 children with SAM who were admitted to the TFU program at Chiro Hospital from September 2019 to August 2020.

#### 2.1.4 Inclusion criteria

This study covered all under‐5 SAM children admitted to the TFU program between September 2019 and August 2020.

#### 2.1.5 Study design and sample size

This study follows a retrospective cohort study design. Due to the relatively small number of SAM cases encountered, a sampling technique was not used. All SAM inpatients who were admitted to the TFU program from September 2019 to August 2020 and met the inclusion criteria were included in this study, resulting in a total sample size of 322 SAM patients.

#### 2.1.6 Data source and data collection procedure

The data source for this study is secondary information obtained from a retrospective cohort study based on hospital records. The data collection period spans from September 2019 to August 2020. Preestablished checklists based on known‐source methodology for the management of SAM were used to collect the data. Data collection involved utilizing the patient monitoring card and registration book from the therapeutic feeding center to gather all essential information.^18^

#### 2.1.7 Study variables

In this research, the response variable is the survival time of SAM under five patients, which is characterized as the interval of time that passes between the diagnosis and one of the following events: “cured,” “defaulted,” “death,” or “transferred out to other health centers or hospitals.” The status variable is entered as 1 for death and 0 for censored, since death is seen as an important occurrence. The predictor variables that are assumed to affect the survival time of children under 5 years of age with SAM include age, residence, sex, pulse rate, respiratory rate, weight‐for‐height, diarrhea, mid‐upper‐arm circumference, vomiting, dehydration, pale conjunctiva, palmar pallor, body temperature, level of consciousness, dermatitis, shock, treatment failure, vitamin A, folic acid, amoxicillin, parenteral antibiotics, intravenous fluid, blood transfusion, nasogastric tube insertion, anemia, malaria, hypoglycemia, tuberculosis, and pneumonia.

### 2.2 Methods of data analysis

#### 2.2.1 Descriptive statistics

Descriptive statistics were used to summarize the variables collected from the patient registration book and study variables for SAM under five children. For this, the frequency distribution table was used.

#### 2.2.2 Survival data analysis

Survival data analysis is suitable for SAM under five data sets commonly observed in medical research. It accounts for right censoring, where patient observations end just before the event of interest. In this study, survival analysis was conducted, and Kaplan‒Meier plots were used to compare the survival functions of different covariate groups. The log‐rank test was employed to determine if there are significant differences in survival times among SAM children under 5 years of age for each covariate.^19^

### 2.3 Definition of some terms

#### 2.3.1 Right censoring

Just before the event, to the right of the last recorded survival time, patient observation ends. Because survival studies generally allow this kind of censoring, it was considered in this study.^20^

Survival time is the time patients started taking the treatment until an event is occurred.

#### 2.3.2 Comparison of survival function

The Kaplan–Meier plots show that the groups of covariates taken into consideration may or may not have different survival periods. However, the log‐rank test was used to ascertain whether the survival time varied for each covariate.^19^ The hypotheses to be tested at 5% level significance are:

For each, the survival curves are identical.

There are differences between the survival curves.

#### 2.3.3 Bayesian survival analysis

The Bayesian approach is recommended for survival analysis due to its ability to combine data analysis with prior knowledge about parameter distributions. It provides more effective analysis than the frequentist approach, particularly for clinical data.^21^ The parameters of the model are viewed as random variables in the Bayesian method and uses prior distributions to account for parameter uncertainty. MCMC techniques, such as the Gibbs sampler, simplify the estimation of complex survival models.^22^ However, MCMC techniques have convergence and computational challenges. The INLA method, introduced in 2009, offers a flexible and rapid approach to approximate the posterior marginal distributions of model parameters.^16^ The INLA method was employed in this study to analyse the SAM data set using Bayesian parametric survival models.

#### 2.3.4 Prior distribution *π*(*θ*)

The prior distribution represents the uncertainty of the parameter before the data are taken into account. It is a probability distribution that takes into account known parameters.^23^

#### 2.3.5 Likelihood function *L*(*θ*|data)

Given the parameters, the likelihood function calculates the likelihood of observing the sample data. For a set of unknown parameters, it can be represented as follows in the presence of right censoring:

$$L\left(\theta /\text{data}\right)=\prod_{j=1}^{n}[f{\left(t_{i}|x_{i};\theta \right)}^{\delta_{i}}*S{\left(t_{i}|x_{i};\theta \right)}^{{1-\delta }_{i}}],$$

where *S* $\left(t_{i}\mathrm{|;}\theta \right)$ and $f\left(t_{i}|x_{i};\theta \right)\;$represent the survival distributions and probability density, respectively, and $\delta_{i}$ is the censoring indicator (1 = death and 0 = censored).^24^ The posterior distribution uses the Bayes rule to integrate the likelihood and prior distribution. A likelihood contains information about model parameters based on the observed data, while a prior contains information about model parameters from before the observed data were observed. To acquire it, multiply the likelihood function by *L*(*θ*|data), which is the prior distribution for all parameters.^25^ Given by

$$\text{Posterior}\;=\frac{\mathrm{Likelihood}\times \mathrm{prior}}{\int \text{Likelihood}\times \text{prior}\;d\theta }.$$

If we assume that is a random variable and that its prior distribution is represented by *π*(*θ*), then the posterior distribution of *θ*, represented by *π*(*θ* | *X*), is as follows:

$$\pi (\theta /X)=\frac{L(X|\theta )\;\times \;\pi (\theta )}{\int L(X|\theta )\;\times \;\pi (\theta )\;d\theta }.$$

The likelihood function *L*(*X*/*θ*) combines the information from the observed data through *L*(*X*|*θ*) with the prior knowledge quantified by *π*(*θ*). This is clear from the fact that the posterior distribution, *π*(*θ* | *X*), is inversely correlated with the likelihood times of the prior, or *π*(*θ* | *X*)∼*L*(*X*|*θ*). The normalizing constant of the posterior distribution, denoted as *m*(*x*), is the integral of the product of the likelihood and the prior, ∫ *L*(*X*|*θ*) × *π*(*θ*)*dθ*. This constant is often referred to as the marginal distribution of the data or the prior predictive distribution. In Bayesian survival analysis, parametric survival models are essential because they offer practical modeling and analytic methods. In Bayesian research, exponential, Weibull, log‐normal, and log‐logistic models are frequently used. These models offer simplicity and ease of use in modeling survival data.^23^ By utilizing parametric survival models, researchers can incorporate prior knowledge and observed data to estimate model parameters and make predictions about survival times. These models provide a framework for understanding the relationship between covariates and survival outcomes, allowing for meaningful interpretation and analysis of survival data in a Bayesian context.

### 2.4 INLA method

To estimate the parameters of Bayesian parametric survival models, one effective method is the INLA method. It has been extensively utilized in the field of survival analysis, which often involves latent Gaussian models. As described in Rue et al.,^16^ INLA allows for the determination of posterior marginals for each component of the model, enabling the derivation of posterior expectations and standard deviations. The latent Gaussian model, combined with INLA, is suitable for modeling survival data. INLA employs innovative Laplace approximations and advanced numerical techniques to generate highly efficient and accurate posterior marginal approximations that are compatible with survival models.^23^ This method provides fast and reliable estimation of the model parameters and allows for insightful analysis of the survival data.

### 2.5 Bayesian model selection criterion

The Deviance Information Criteria could be used to compare Bayesian parametric survival models (DIC). The model with the lowest DIC value should be used.^26^ The Watanabe Akaike Information Criteria (WAIC),^27^ which offers a criterion utilizing a more deeply Bayesian methodology, is an alternative.^17^ Claims that the WAIC is better than the DIC.

### 2.6 Bayesian model diagnostics

The two most widely used techniques for assessing goodness of fit are the predictive distribution and the Bayesian Cox‐Snell residual plot. Models for survival data must include both model checking and model adequacy. The Bayesian analysis used shows the residuals' Bayesian representation.^28^

## 3 RESULTS

### 3.1 Descriptive statistics

The survival endpoint of interest in this study was death. Therefore, the date of the first entry was subtracted from the date of the last date (the death date) to construct the time‐to‐death, or death time in days. Accordingly, of the 322 SAM children under the age of five, 118 (36.6%) died, according to statistics collected from the Chiro hospital, while the remaining 204 (or 63.4%) were censored. According to the SAM management protocol, the patient's tolerable survival time in the therapeutic feeding facilities is 2 weeks, or a median survival time of 14 days, with an interquartile range of 1.2 to 3.3 weeks, as shown in Table 1.

**Table 1:** Survival status and median survival time for children under 5 years old with severe acute malnutrition from September 2019 to August 2020 at Chiro Hospital.

Censored	Event	Total	Median survival time	[95% Conf. Interval]	
				LCL	UCL
204 (63.4%)	118 (36.6%)	150	2 weeks	1.2 weeks	3.3 weeks

The results of the categorical predictor variables for SAM in children under 5 years of age are shown in Table 2 below. A total of 189 (58.7%) of the 322 SAM children under the age of five were female. Out of a sample of 322 SAM children under the age of five, 125 (38.8%) lived in a rural location. Out of a sample of 322 SAM children under the age of five, 153 (47.5%) of the inpatients were older than 24 months. Out of a sample of 322 SAM children under the age of five, 184 (57%) had no diarrhea.

**Table 2:** Descriptive results of categorical variables of SAM children under the age of five, Chiro hospital, September 2019 to August 2020.

Factors		Survival status		Total (%)
		Censored 204 (63.4%)	Event 118 (36.6%)	
Residence	Rural	72 (22.7%)	53 (16.1%)	125 (38.8%)
	Urban	130 (40.7%)	67 (20.5%)	197 (61.2%)
Sex	Female	127 (39.1%)	62 (19.6%)	189 (58.7%)
	Male	78 (24.2%)	55 (17.1%)	133 (41.3%)
Age category	>24 months	127 (39.4%)	26 (8.1%)	153 (47.5%)
	≤24 months	77 (23.9%)	92 (28.6%)	169 (52.5%)
WT/HT	≥70%	151 (46.9%)	38 (11.8%)	189 (58.7%)
	<70%	53 (16.5%)	80 (24.8%)	133 (41.3%)
Diarrhea	No	163 (50.6%)	21 (6.4%)	184 (57%)
	Yes	41 (12.7%)	97 (30.3%)	138 (43%)
MUAC	≥11.5	106 (32.9%)	25 (7.8%)	131 (40.7%)
	<11.5	98 (30.4%)	93 (28.9%)	191 (59.3%)
Vomiting	No	93 (28.9%)	18 (5.6%)	111 (34.5%)
	Yes	96 (29.8%)	115 (35.7%)	211 (65.5%)
Type of SAM	No edematous	153 (47.5%)	21 (6.5%)	174 (54%)
	Edematous	51 (15.8%)	97 (30.2%)	148 (46%)
Respiratory rate	Normal	150 (46.58%)	21 (6.5%)	171 (53.11%)
	Altered	54 (16.77%)	97 (30.2%)	151 (46.89%)
Dehydration	No	186 (57.76%)	52 (16.14%)	238 (73.9%)
	Yes	18 (5.6%)	66 (20.5)	84 (26.1%)
Pulse rate	Normal	170 (52.8%)	80 (24.8%)	250 (77.6%)
	Altered	34 (10.6%)	38 (11.8%)	72 (22.4%)
Pale conjunctiva	No	127 (39.4%)	26 (8.1%)	153 (47.5%)
	Yes	77 (23.9%)	92 (28.6%)	169 (52.5%)
Palmar pallor	No	153 (47.5%)	21 (6.5%)	174 (54%)
	Yes	51 (15.8%)	97 (30.2%)	148 (46%)
Body temperature	Normal	106 (32.9%)	25 (7.8%)	131 (40.7%)
	Altered	98 (30.4%)	93 (28.9%)	191 (59.3%)
Level of consciousness	Normal	8 (2.5%)	40 (12.4%)	48 (14.9%)
	Altered	196 (60.9%)	78 (24.2%)	274 (85.1%)
Dermatitis	Present	73 (22.7%)	52 (16.1%)	125 (38.8%)
	Absent	131 (40.68%)	66 (20.5%)	197 (61.18%)
Shock	No	186 (57.76%)	52 (16.14%)	238 (73.9%)
	Yes	18 (5.6%)	66 (20.5)	84 (26.1%)
Treatment failure	No	126 (39.13%)	37 (11.5%)	163 (50.62%)
	Yes	70 (21.74%)	89 (27.64%)	159 (49.38%)
Vitamin A	No	120 (37.27%)	43 (13.35%)	163 (50.62%)
	Yes	70 (21.74%)	89 (27.64%)	159 (49.38%)
Folic acid	No	120 (37.27%)	43 (13.35%)	163 (50.62%)
	Yes	67 (20.81%)	92 (28.57%)	159 (49.38%)
Amoxicillin	No	73 (22.7%)	52 (16.1%)	125 (38.8%)
	Yes	121 (37.58%)	76 (23.6%)	197 (61.18%)
Parenteral antibiotic	No	150 (46.58%)	24 (7.45%)	174 (54%)
	Yes	50 (15.53%)	98 (30.43%)	148 (46%)
IV fluid	No	126 (39.13%)	37 (11.5%)	163 (50.62%)
	Yes	70 (21.74%)	89 (27.64%)	159 (49.38%)
Blood	No	129 (40.06%)	34 (10.56%)	163 (50.62%)
	Yes	70 (21.74%)	89 (27.64%)	159 (49.38%)
NG tube insertion	No	143 (44.41%)	31 (9.63%)	174 (54%)
	Yes	51 (15.8%)	97 (30.2%)	148 (46%)
Anemia	No	151 (46.9%)	38 (11.8%)	189 (58.7%)
	Yes	39 (12.11%)	99 (30.74%)	138 (43%)
Malaria	No	151 (46.9%)	38 (11.8%)	189 (58.7%)
	Yes	49 (15.22%)	84 (26.08%)	133 (41.3%)
Hypoglycemia	No	93 (28.9%)	18 (5.6%)	111 (34.5%)
	Yes	90 (27.95%)	121 (37.58%)	211 (65.5%)
Tuberculosis	No	127 (39.44%)	26 (8.07%)	153 (47.52%)
	Yes	70 (21.74%)	99 (30.74%)	169 (52.48%)
Pneumonia	No	83 (25.78%)	52 (16.1%)	135 (41.92%)
	Yes	121 (37.58%)	66 (20.5%)	187 (58.07%)

Abbreviations: MUAC, mid‐upper arm circumference; NG, nasogastric; SAM, severe acute malnutrition.

**Figure 1:**
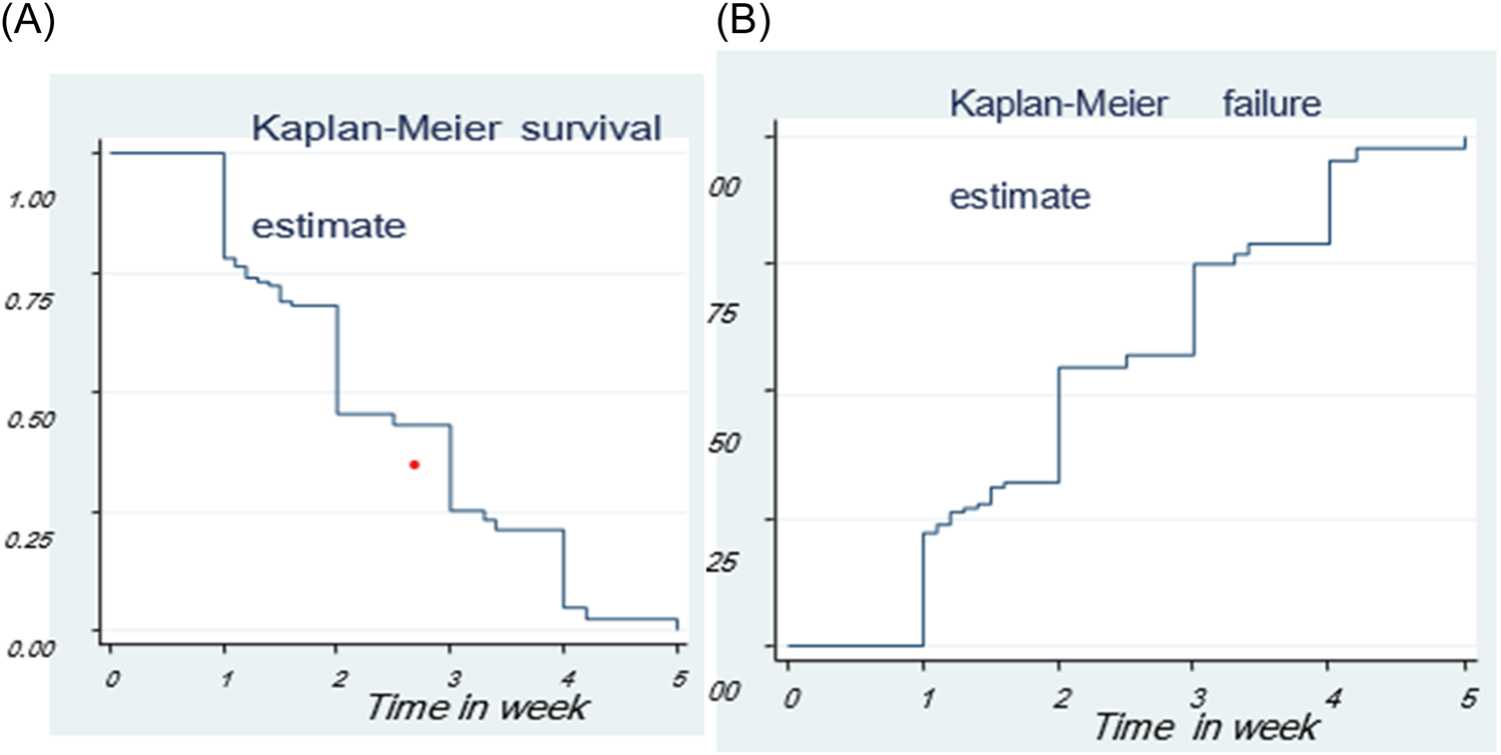
(A) The K‒M curve for survival function. (B) K‒M curve for hazard functions for severe acute malnutrition of underfive children data set, Chiro hospital, September 2019 to August 2020.

Figure 1A displays the survival time from SAM in children under the age of five. The probability of surviving (*p*(*T* > *t*)) is shown by the vertical axis, while the horizontal axis shows the survival time. At the beginning, the survival curve shows an increasing trend, indicating a high probability of recovery for children in the early stages of SAM. The curve starts at *t* = 0 with *S*(*t*) = 1, representing a complete probability of survival. However, as time progresses, the survival curve starts to decrease, indicating a decline in the likelihood of recovery for children under 5 years of age with SAM. Figure 1B presents a hazard plot, where the survival time from SAM in underfive children is shown on the horizontal axis, and the cumulative hazard is shown on the vertical axis. The curve illustrates that the hazard of underfive children with SAM increases as the analysis time increases. This implies that the risk of experiencing an event, such as mortality or worsening health conditions, becomes higher as time passes.

### 3.2 Kaplan‒Meier estimates of some independent variables

**Figure 2:**
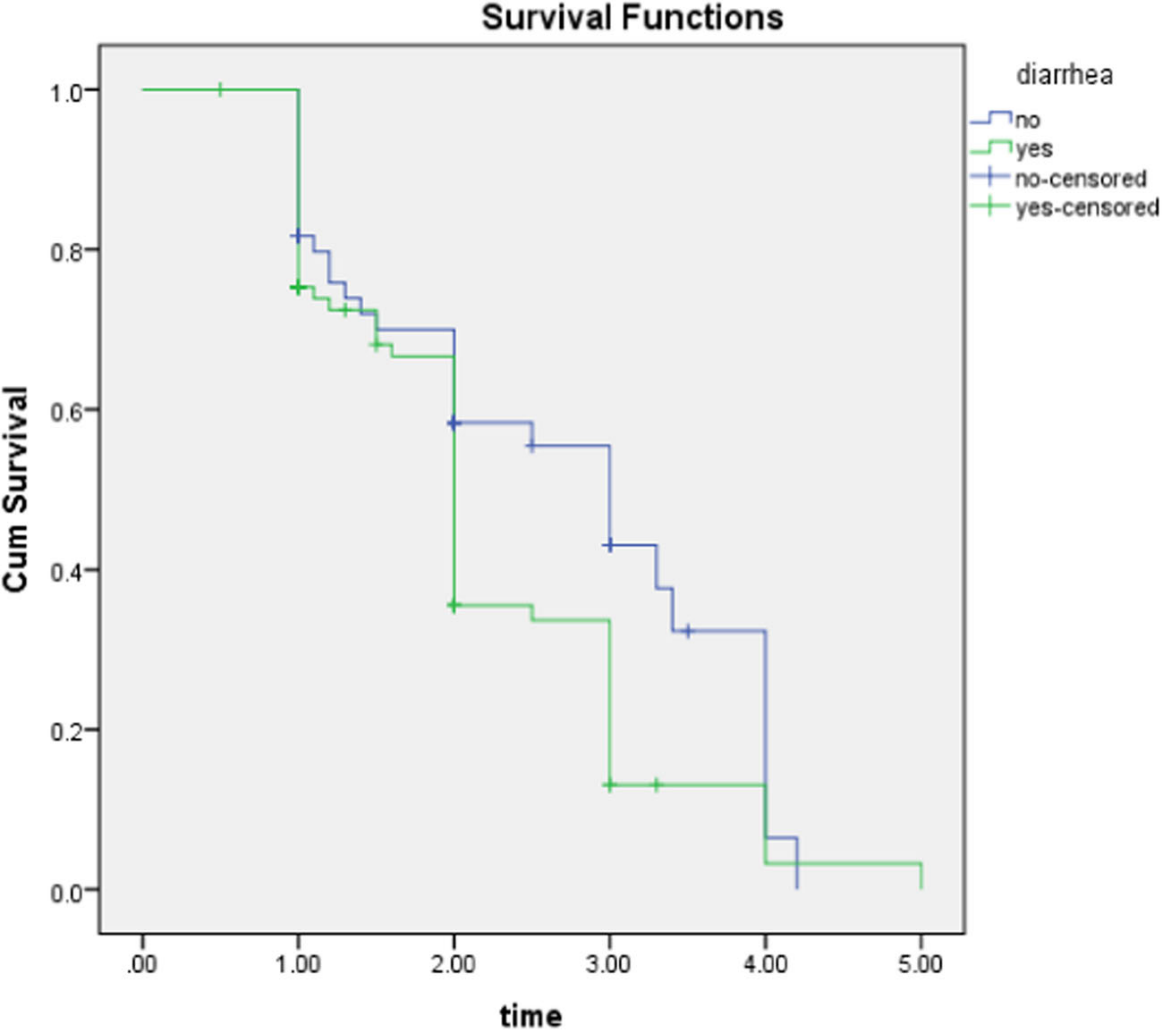
Kaplan‒Meier plot of diarrhea at Chiro Hospital from September 2019 to August 2020.

In Figure 2, the survival curves of children under 5 years old with SAM are compared based on the presence of diarrhea. This comparison demonstrates that the presence of diarrhea has an impact on the probability of survival. The curves show variations throughout the study period, indicating that the presence of diarrhea influences the survival outcomes for children with SAM.

### 3.3 Checking Cox PH assumption and variable selection

The analysis revealed that the Cox‐PH model assumption was not valid for the SAM data set of children under the age of five. The *p* values for the variables pneumonia and hypoglycemia were found to be smaller than the typical significance level of 5%. Additionally, the correlation between Schoenfeld residuals and survival time was statistically significant, as shown in Table 3. This indicates that the assumption of proportional hazards was violated in the Cox‐PH model for the SAM under five children data set. Alternative parametric accelerated failure time (AFT) models were used to overcome this problem. Multivariable analysis using Bayesian AFT models with the INLA method was conducted. The time variable “ti” represents the survival time for SAM under five children, where “*i*” ranges from 1 to 322 in the data set. The covariate coefficients, denoted as *β* = (*β*0, *β*1…, *β*p)’, including the intercept (*β*0) and *p*−1 additional covariates, were assumed to have a normal prior with a mean of 0 and a variance of 1000. A gamma prior with a shape parameter of 1 and an inverse scale parameter of 0.001 was used for the Weibull, log‐normal, and logistic distributions.^28^ Table 4 presents the comparison of models using the SAM under five children data set. The different parametric AFT models were assessed, and their performance was evaluated based on the data set. The most suitable model for analysing the survival time of SAM under five children can be chosen thanks to this analysis. Based on the acceleration factor and 95% credible interval of the predicted Bayesian accelerated failure time values from Table 5, the resulting model was interpreted. The estimated acceleration factor is denoted by the formula *γ* = [exp($\hat{\beta }$)] = [exp(posterior mean)].

**Table 3:** Cox model's proportional hazard assumption for SAM in 5‐year‐old children at Chiro Hospital from September 2019 to August 2020.

Covariate	Chi‐square	*Df*	*p* Value
Pulse rate	0.41	1	0.6
Shock	0.12	1	0.90
NG tube	1.2	1	0.3
Anemia	0.7	1	0.40
Hypoglycemia	3.9801	1	0.05
Vitamin A	1.0028	1	0.32
Age	2.7651	1	0.46
Body temperature	0.1897	1	0.66
Anemia	0.0772	1	0.78
Dehydration	0.032	1	0.45
Respiration rate	0.44	1	0.06
Antibiotic	0.094	1	0.08
Pneumonia	4.087	1	0.05
diarrhea	0.98	1	0.12
MUAC	0.57	1	0.68
GLOBAL	26.2570	15	0.05

Abbreviations: MUAC, mid‐upper arm circumference; NG, nasogastric; SAM, severe acute malnutrition.

**Table 4:** Comparisons of the Bayesian AFT model using INLA methods, SAM in 5‐year‐old children, Chiro Hospital, September 2019 to August 2020.

Distributions	DIC	WAIC
Exponential	1504.82	1636.63
Log‑Normal	1491.34	1492.55
**Weibull**	**1399.84**	**1398.77**
Log‐logistic	1430.91	1430.67

Abbreviations: AFT, accelerated failure time; Deviance Information Criteria; INLA, integrated nested Laplace approximation; SAM, severe acute malnutrition; WAIC, Watanabe Akaike Information Criteria.

*Bold values indicate better results than other filtering methods.

**Table 5:** Bayesian AFT model using INLA methods Results of SAM under 5 age children, Chiro hospital, September 2019 to August 2020.

Parameter		Pmean	SD	Median	Credible interval	Mode	Kld
	Intercept	5.763	0.191	4.8	[5.45, 6.51]*	5.70	0
Age	ref = ≥24 months						
	<24 months	−0.401	0.215	−0.401	[−0.632, −0.175]*	−0.399	0
Body temperature	ref = Normal						
	Altered	−0.168	0.025	−0.166	[−0.398, −0.027]*	−0.163	0
Antibiotic	ref = No						
	Yes	−0.158	0.113	−0.16	[− 0.382, 1.143]	−0.16	0
Pulse rate	Ref = Normal						
	Altered	−0.435	0.100	−0.434	[−0.656, −0.224]*	−0.430	0
MUAC	ref = ≥11.5						
	<11.5	−0.168	0.123	−0.168	[−0.392, 0.073]	−0.167	0
Shock	ref = No						
	Yes	−0.279	0.062	−0.278	[−0.427, −0.134]*	−0.277	0
NG tube insertion	ref = No						
	Yes	−0.201	0.016	−0.201	[−0.442, − 0.053]*	−0.200	0
Anemia	ref = No						
	Yes	−0.282	0.05	−0.270	[−0.389, −0.06]*	−0.279	0
Hypoglycemia	ref = No						
	Yes	−0.243	0.072	−0.232	[−0.289, −0.028]*	−0.231	0
Respiration rate	ref = Normal						
	Altered	−0.198	0.153	−0.128	[−0.482, 1.243]	−0.167	0
Vitamin A	ref = No						
	Yes	−0.257	0.213	−0.358	[−0.292, 1.643]	−0.557	0
Diarrhea	ref = No						
	Yes	−0.332	0.11	0.331	[−0.531, −0.136]*	−0.330	0
Dehydration	ref = No						
	Yes	−0.205	0.120	−0.205	[−0.428, −0.098]*	−0.214	0
Malaria	ref = No						
	Yes	−0.168	0.143	−0.158	[−0.282, 1.063]	−0.157	0
Pneumonia	ref = No						
	Yes	−0.327	0.063	−0.126	[−0.400, −0.017]*	−0.124	0
Tau parameter (Weibull)							
		4.19	0.497	4.18	[3.29, 5.27]*	4.15	_

Abbreviations: MUAC, mid‐upper arm circumference; NG, nasogastric; Pmean, posterior mean; SAM, severe acute malnutrition; SD, standard deviation.

* indicates statistical significance at 5%.

The WAIC and Deviance Information Criteria (DIC) were used to compare the effectiveness of various models. The model that is thought to best suit the data has the smallest DIC and WAIC scores. In this case, the Bayesian Weibull AFT model showed the lowest DIC (1399.93) and WAIC (1398.67) values, indicating that it provided the best prediction of the survival time for SAM children under the age of five. After selecting the Bayesian Weibull AFT model, a multivariable analysis was conducted using the significant covariates determined from the univariate analysis and the purposeful variable selection strategy (at a significance level of 25%). The final results of the Bayesian Weibull AFT model are presented in Table 5, showing the estimated values and statistical significance of factors such as age, body temperature, pulse rate, NG tube, hypoglycemia, anemia, diarrhea, dehydration, malaria, and pneumonia on the survival times of SAM children under the age of five. The acceleration factor and the 95% credible range of the Bayesian accelerated failure time predicted values can be used to analyse the resultant model. The acceleration factor, denoted as *γ*, is estimated as the exponential of the posterior mean of the coefficients (*β̂*). The acceleration factor represents the change in the survival time associated with a one‐unit change in the corresponding covariate.

Based on the estimated values from the Bayesian Weibull AFT model, while keeping the effect of other factors constant, the estimated acceleration factor for SAM under five children who are less than 24 months old is 0.77. The 95% credible interval (CrI) for this acceleration factor ranges from −0.486 to −0.035. This indicates that age has a statistically significant effect on the survival time of SAM under five children. Specifically, for each one‐unit increase in age (in months), the survival time is expected to decrease by a factor of 0.77, on average, when considering the impact of other factors in the model. The credible interval suggests that this effect is statistically significant and provides a range of plausible values for the true acceleration factor. SAM under‐five children with an altered pulse rate have an estimated acceleration factor of 0.65, with a 95% credible interval of 0.52–0.80. This suggests that the expected survival time decreases by 35% for SAM under five children with an altered pulse rate compared to those with a normal pulse rate. SAM under five children with diarrhea had an estimated acceleration factor of 0.72, with a 95% credible interval of 0.59‐0.87. This indicates that the expected survival time is 18% shorter for children with SAM under 5 years with diarrhea than for those without diarrhea. In Table 5, all significant parameters in the Bayesian Weibull AFT model have Kullback‒Leibler divergence values of 0. The posterior distribution closely resembles a normal distribution if there is a low divergence value. The simplified Laplace approximation was the fastest and most effective approach for this analysis, providing accurate results.

### 3.4 Bayesian model diagnostic

**Figure 3:**
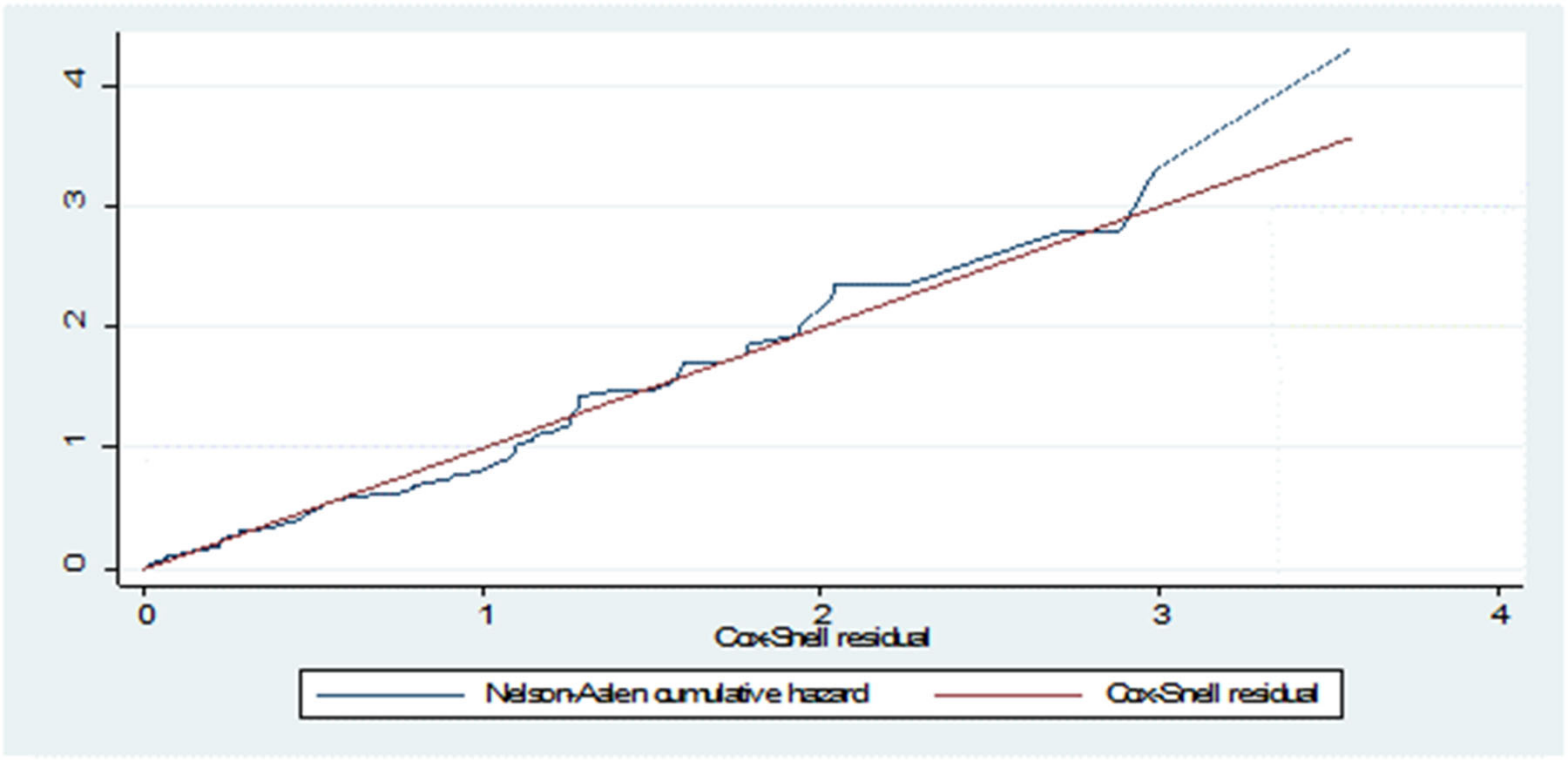
Bayesian Cox‐Snell residual plots for baseline distribution and Cox‐PH that were used to fit the severe acute malnutrition under the 5‐year‐old children data set, Chiro Hospital, September 2019 to August 2020.

A satisfactory match was shown by the Bayesian Cox‐Snell residual plot for the Bayesian Weibull AFT model, which showed a close alignment with the line across the origin. The model successfully captured the relationship between the residuals and the cumulative hazard function, as shown by the Cox‐Snell residual plot versus the cumulative hazard function of residuals, which showed a straight line with a slope of 1. Figure 3, which depicts the Bayesian Cox‐Snell residual plots, further supports the suitability of the Bayesian Weibull AFT model for the SAM under five age children data set. The plots illustrate how well the model describes the data and its ability to capture the underlying survival patterns in children under the age of five. The graphs presented 95% confidence intervals and were based on the posterior density for the parameters assumed to be normally distributed in the SAM under five age children data set. The Kullback‒Leibler divergence (kld) values in Table 5 indicated that the INLA approximation provided accurate results, as the kld values were all zero for the relevant parameters in the Bayesian Weibull AFT model.

## 4 DISCUSSIONS

This study aimed to identify variables that influence the survival times of children with SAM under the age of five at Chiro Hospital. According to the study's descriptive statistics, 322 SAM children under the age of five were involved in the study overall. According to the SAM management protocol, the patient's acceptable survival time in the therapeutic feeding facilities is 2 weeks, or a median survival time of 14 days, with an interquartile range of 1.2–3.3 weeks. An analysis using Bayesian parametric survival modeling was performed on this set of data. However, the fundamental premise of the Cox‐PH model was flawed. The Bayesian technique was utilized to assess the effectiveness of several AFT models, and DIC and WAIC were computed.^7^, ^29^ The Bayesian Weibull AFT model demonstrated the best performance among several parametric models, including Cox‐PH, in predicting survival time. Findings from a previous study^30^ led to similar results. The findings indicated that age, body temperature, pulse rate, NG tube usage, hypoglycemia, anemia, diarrhea, dehydration, malaria, and pneumonia significantly affected the survival time of SAM children under five. The study revealed that out of the 322 SAM children included in the analysis, 118 (36.6%) died, which was higher compared to previous studies conducted in Ethiopia. Out of 346 patients, 223 (64.4%) died.^10^ Younger age (<24 months) was associated with a 33% shorter expected survival time, and younger children were at a higher risk of mortality due to lower immunity, increased vulnerability to illnesses, and inadequate feeding practices. This was consistent with Jarso et al.'s research,which indicated that they had a twofold increased risk of passing away earlier.^31^ The findings also coincide with different studies,^32^ but age is not a predictor of survival time.^7^ This discrepancy may have resulted from enrollment of the most vulnerable age groups (those under 6 months), which had the highest death risk. Among SAM children under the age of five, anemia was discovered to be a highly significant predictor of lower survival time. SAM children with anemia had a 25% shorter expected survival time than those without anemia. According to a study performed in a hospital in Sekota, in northern Ethiopia, the case fatality rate for severe anemia is also higher.^7^ Similarly, studies conducted in South Africa and Niger using the same measurement showed that the risk of mortality is higher in exposed children.^29^ In Jimma University Specialized Hospital, the probability of death for children with anemia was 2.1 times higher (assessed by palmar pallor).^31^ This is because anemia makes infections more common, heart failure more likely, and compliance will generally decline. Altered body temperature (hypothermia and hyperpyrexia) was also a significant predictor, with SAM children experiencing altered body temperature having a 16% shorter expected survival time. Children with changed body temperatures were seven times as likely to die sooner than kids with normal temperatures. In another study, hypothermia tripled the risk of mortality,^31^ although in contrast to these results, a South African investigation found no association.^29^ The mortality associated with such modification is substantial because hypothermia and hyperpyrexia alter the body's metabolic processes and are signs of altered metabolism, sepsis, and severe infections.^33^

Pulse rate and the presence of shock were identified as significant factors affecting survival time. SAM children with an altered pulse rate had a 35% shorter expected survival time than those with a normal pulse rate. Hypoglycemia was associated with a 22% decrease in expected survival time for SAM children under five. The use of an NG tube for feeding was another significant predictor, with children fed through an NG tube experiencing an 18% shorter expected survival time and a higher mortality rate. According to a study, children's mortality risk increased by 3.9 times when there was an undetectable pulse present.^34^ A dismal prognosis results when the illness proceeds to shock; altered pulse rate is a main sign of shock, acute infections, severe dehydration, and fluid and electrolyte imbalance, all of which are fatal.^35^ According to the study's findings, hypoglycemia was a significant predictor of survival time for SAM children under the age of five. The expected survival time of hypoglycemic children decreases by 22% compared to normoglycemic SAM children under 5. The findings of this study, which indicated that hypoglycemia is a risk factor for poor outcome in children who are severely malnourished, were consistent with those previously reported by other authors^36^; however, they diverged from those of a Ugandan study, which concluded that there was insufficient proof to link hypoglycemia with an increased risk of death.^37^ The study's findings showed that among children with SAM under the age of five at Chiro Hospital, the NG tube was a highly significant predictor of survival. The expected survival time of children fed with NG tubes decreased by 18% compared to that of children fed per mouth. Children fed using an NG tube had a three times higher mortality rate than those fed through the mouth. An NG tube being needed when a child has altered awareness, shock, or a serious infection may help to explain this. Additionally, issues such as aspiration, which partially contribute to high mortality, and failed appetite tests may also be to blame.^32^

## 5 LIMITATIONS OF THE STUDY

The study acknowledged limitations such as the use of secondary data, which may have introduced inaccuracies or biases. The absence of certain variables in the medical records was also noted as a limitation, and future research could consider including those variables to enhance the analysis.

## 6 CONCLUSIONS AND RECOMMENDATION

The survival rates of SAM children under the age of five were taken from a patient data set that was being treated at Chiro Hospital for this study. Several parametric models with baseline distributions (log‐logistic, Weibull, exponential, and log‐normal) were outperformed by the Bayesian Weibull AFT model. DIC and WAIC show that these models perform well in terms of prediction. As a result, they may be considered trustworthy tools for prognostic prediction, which is essential for raising the patient's chances of survival. Cumulative hazard graphs and diagnostic charts confirmed the suitability of the Bayesian Weibull AFT model for the SAM children under five data sets. The model demonstrated good predictive performance and accuracy. Additional research by Akerkar and colleagues^23^, ^38^ confirmed this conclusion. The findings of this study suggested that <24 age, altered body temperature, altered pulse rare, NG tube, hypoglycemic children, SAM children with anemia, SAM children with diarrhea, dehydration, SAM children with malaria and SAM children with pneumonia all shortened the survival time of SAM children under 5. In conclusion, the study recommended early initiation of therapy for all SAM children under five, particularly high‐risk groups such as those with malaria and younger age. To reduce the death rate of children under 5 years of age, it is necessary to design community management for acute malnutrition to ensure early detection and improve access to and coverage for children who are malnourished. Strengthening screening programs and improving the chances of survival for SAM children under five were also recommended.

## AUTHOR CONTRIBUTIONS

**Chalachew Gashu**: Conceptualization; methodology; formal analysis; data curation; writing—original draft; investigation; software; writing—review and editing; validation. **Yoseph Kassa**: Conceptualization; investigation; writing—review and editing; data curation. **Habtamu Geremew**: Methodology; writing—original draft; conceptualization; investigation; project administration. **Mengestie Mulugeta**: Methodology; conceptualization; investigation; writing—original draft; formal analysis.

## CONFLICT OF INTEREST STATEMENT

The authors declare no conflict of interest.

## ETHICS STATEMENT

The Oda Bultum University, College of Natural & Computational Science, ethical approval committee (04/03/976/10/2015) provided the letter of permission for ethical clearance. Written informed consent was obtained because there were no SAM children under 5 at the Chiro hospital when the data were collected. The use of written informed consent, obtained over the phone from study participants, was accepted by both institutions. The patient card contained the caregiver's phone number, which we used for those research participants who passed away. They provided written, informed consent. Patients' cards for those who agreed to take part in the study were retrospectively examined from September 2019 to August 2020 to gather data on the sociodemographics, medical histories, and therapies received by outpatients. The data collectors informed participants about the study's goal and purpose.

## TRANSPARENCY STATEMENT

The lead author Chalachew Gashu affirms that this manuscript is an honest, accurate, and transparent account of the study being reported; that no important aspects of the study have been omitted; and that any discrepancies from the study as planned (and, if relevant, registered) have been explained.

## Data Availability

The data that support the findings of this study are available from the corresponding author upon reasonable request. Because the data were used for another investigation, the authors affirm that the data supporting the study's findings are not publicly available. On reasonable request, the corresponding author does, however, provide data. Each author has reviewed and approved the completed paper. They all had complete access to all study data and are solely accountable for the accuracy and integrity of the data analysis.
